# Risky sexual networks and concentrated HIV epidemics among men who have sex with men in Wenzhou, China: a respondent-driven sampling study

**DOI:** 10.1186/s12889-015-2591-7

**Published:** 2015-12-16

**Authors:** Qiaoqin Ma, Shidian Zeng, Shichang Xia, Xiaohong Pan, Dayong Wang, Haishen Zhu, Hui Wang, Tingting Jiang, Lin He, Dongshe Zhao, Zhihang Peng

**Affiliations:** Department of HIV/STD Control, Center for Disease Control and Prevention of Zhejiang Province, No.3399, Binsheng Road, Hangzhou, 310051 People’s Republic of China; Center for Disease Control and Prevention of Wenzhou Municipality, Wenzhou, 3250051 People’s Republic of China; Center for Disease Control and Prevention of Lucheng District, Wenzhou, 325001 People’s Republic of China; Nanjing Medical University School of Public Health, Nanjing, 211166 People’s Republic of China

**Keywords:** HIV, Men who have sex with men, Sexual network, China

## Abstract

**Background:**

The high and continually increasing prevalence of human immunodeficiency virus (HIV) and other sexually transmitted diseases among men who have sex with men (MSM) in China underscores the critical importance of examining the exact sexual networks that result in HIV transmission, as well as HIV infection, using powerful sampling methods, such as respondent-driven sampling (RDS), to improve the sexual health of this population.

**Methods:**

Using RDS, a cross-sectional study was conducted among MSM in Wenzhou, Zhejiang province, China from December 2013 to June 2014. The type of sex, numbers of anal sex partners, male oral sex partners and vaginal sex partners, condom use during each type of sex over the previous 6 months, prevention behaviors, risk perception, and the burdens of HIV and syphilis were investigated and analyzed.

**Results:**

Of 424 MSM, a great number of them did anal sex, male oral sex, and vaginal sex during the previous 6 months, and weighted estimates for the prevalence that MSM did not conduct these sexual behaviors were 11.2 % (95 % confidence interval [CI] =6.7–16.50 %), 20.3 % (95 % CI = 15.2–27.1 %), and 58.9 % (95 % CI = 52.1–65.8 %), respectively. Multiple sexual partners, engaging in regular, casual and commercial sex, and lack of condom use during all types of sex were common among MSM. The estimated HIV and syphilis prevalences were 22.8 % (95 % CI = 16.9–28.5 %) and 9.7 % (95 % CI = 6.4–13.6 %), respectively. Of the participants, 53.5 % (95 % CI = 45.3–60.2 %) received HIV-related interventions during the previous year, 48.1 % (95 % CI = 39.7–55.1 %) had never been tested for HIV, and only 14.1 % (95 % CI =10.1–19.2 %) perceived a risk of contracting HIV. Multiple logistic regression analysis revealed that age over 44 years (adjusted odds ratio [AOR] = 3.60, 95 % CI = 1.34–9.64), a monthly income of 3001–4000 yuan (approximately 470–630 US$) (AOR = 1.96, 95 % CI = 1.67–3.60), multiple anal sex partners (AOR = 1.93, 95 % CI = 1.15–3.24), awareness of the possibility of contracting HIV (AOR = 3.18, 95 % CI = 1.56–6.48), and current syphilis infection (AOR = 3.01, 95 % CI = 1.44–6.29) were predictors of HIV infection.

**Conclusions:**

HIV transmission has become highly prevalent and will likely become more prevalent among MSM and their female partners if these risky sexual networks persist. Our findings call for urgent and effective interventions to prevent the rapid transmission of HIV among MSM in Wenzhou.

**Electronic supplementary material:**

The online version of this article (doi:10.1186/s12889-015-2591-7) contains supplementary material, which is available to authorized users.

## Background

In China, men who have sex with men (MSM) are at high-risk for HIV. The rate of homosexual transmission among annually reported human immunodeficiency virus (HIV) cases increased from 2.5 % in 2005 to 25.8 % in 2014 [1, 2]. Of the estimated 48,000 new infection cases in 2011, homosexual transmission accounted for 29.4 % [1]. The prevalence of HIV among MSM in China was 8.0 % in four cities from 2008 to 2009 [3]. Several studies using respondent-driven sampling (RDS) showed a HIV prevalence of 5–8 % among MSM [4–6], and this prevalence is increasing rapidly. According to national sentinel surveillance data, the HIV prevalence was 0.9 % in 2003, increasing to 6.3 % in 2011 [1]. In Guangzhou, the HIV prevalence increased from 5.0 % in 2008 to 11.4 % in 2013 [7], and in Chongqing it increased precipitously from 11.6 % in 2009 to 15.4 % in 2010 [8]. Several prospective studies have revealed an upward trend in HIV incidence among MSM in different parts of China [9, 10].

Epidemiological studies have also indicated a high prevalence of syphilis among MSM, ranging from 9 % to 18 % in various populations [3, 5, 11, 12]. A meta-analysis indicated that the syphilis prevalence among MSM in China increased from 6.8 % in 2003–2004 to 10.4 % in 2005–2006 and to 13.5 % in 2007–2008 [13].

The high and increasing prevalences of HIV and syphilis among MSM underscore the critical importance of examining sexual activities that increase the risk of HIV and syphilis transmission. Risky sexual behaviors including multiple homosexual partners, concurrent sex with women, and poor condom use have been reported previously in Chinese literature [4, 6, 14–17]. However, few studies in China have systematically examined the frequency of different sexual behaviors and the characteristics of sexual networks, let alone used a powerful sampling method to pinpoint the current dynamics of HIV transmission, thereby optimizing future prevention activities to decrease the rate of HIV transmission in the study population.

This study was conducted in Wenzhou, which is located on the east coast of China and is among the three largest cities in Zhejiang Province. It covers an area of 11,784 km^2^ with a population of 9.122 million [18]. Wenzhou, a developed and commercialized city, is famous for its entrepreneurial spirit and prosperous private economy and has attracted people from different parts of the country for jobs and opportunities. Of its residential population, 2.842 million have moved from other areas [18]. There is a serious HIV epidemic among MSM in Wenzhou, with a rapid increase in HIV prevalence from 1.5 % in 2008 to 7.2 % in 2013, and from 1.5 % to 8.5 % during this same period in Zhejiang province as a whole [19]. The aim of this study was to explore the behaviors related to anal sex, oral sex with male partners, and sex with female partners and the HIV-related risks among MSM, thereby enabling interventions to improve the sexual health of this population.

## Methods

### Study setting and participants

This research was conducted from December 2013 to June 2014. The MSM sites included one gay bar, one bathhouse, and two public parks in Wenzhou. The inclusion criteria in this study were an age of at least 14 years old, residence in Wenzhou for at least 3 months, self-reports of anal sex and/or oral sex with at least one man in the previous 12 months, and willingness to participate in the survey. Lucheng District Center for Disease Control and Prevention (CDC), which is located in the downtown area of Wenzhou and has a good relationship with the MSM community, was selected as our sole research site to avoid the possibility of recruiting a subject more than once.

### Respondent-driven sampling and data collection

Respondent-driven sampling (RDS) was employed to recruit the participants. RDS is widely used as a sampling method for recruiting hidden and stigmatized populations such as MSM and for collecting data on social network size, and the results obtained from RDS are considered to be generalizable to the entire population [20–22]. In brief, recruitment starts with identification of potential ‘seeds’, who are then asked to recruit their peers, who in turn refer others. The sample composition reaches equilibrium within a number of recruitment waves, regardless of the characteristics of the initial seeds [20]. The sample composition and characteristics are stable after reaching equilibrium and will not change much with subsequent recruitment of peers.

Based on recommendations from local MSM communities and Lucheng CDC, five initial eligible participants identified as being popular among MSM were chosen as seeds. Each seed recruited a maximum of three participants, who then recruited another three participants, and so forth. All participants received three coupons to recruit members from their social network. Each coupon was coded to link their survey responses and monitor who recruited whom; the address, phone number, and office hours of the research site were also listed. The recruitment continued day and evening without break during the research period. In this study, each of the five seeds recruited 30, 61, 81, 103, and 174 MSM, respectively, for a total of 454 subjects. Equilibrium was reached by wave 14 regarding socio-demographic distributions (age, education, marital status, and monthly income) and prevalence of HIV and syphilis. The average network size was seven members. Each participant was offered a monetary compensation of 100 Yuan (approximately 16 US$[United States dollars]) for their participation and peer referral.

Out of the 454 participants, 424 who partook in anal or oral sex during the previous 6 months and responded to all of the study questions were included in the analysis.

The questionnaire used Additional file 1 was developed based on reviews of various domestic and international studies and repeated discussion among members of the research team, local CDC, and MSM communities. It was then piloted among MSM for its wording and structure. Participants were briefed in detail about the purpose and methods of the study, and it took approximately 20–30 min to complete the interview. All enrolled participants were interviewed by the trained staff of Lucheng CDC, who also filled in the questionnaire.

### HIV and syphilis testing

The participants underwent HIV and syphilis testing after the interview. A venous blood sample (5 ml) was collected and subjected to a rapid HIV test at Lucheng CDC, followed by enzyme-linked immunosorbent assay (ELISA) to confirm positive results. The presence of HIV antibodies was confirmed by Western blotting at Wenzhou municipal CDC. Samples were tested for syphilis using ELISA; positive results were confirmed by the rapid plasma reagin test or toluidine red unheated serum test. Participants who tested positive for HIV and/or syphilis were informed and counseled by the staff of Lucheng CDC, and referral services were provided to these cases.

### Ethical considerations

The ethical committee of Zhejiang provincial CDC reviewed and approved the procedures and the protocol of this study. No harm or risk was presented to the participants, and the purpose of the study was to improve the health of MSM in Wenzhou. Participant confidentiality and anonymity were strictly preserved. Written informed consent was obtained before commencement of the survey.

### Statistical analysis

Due to the sampling method, individuals with larger social networks were more likely to be recruited, and thus the data were adjusted for the size of each participant’s social network (based on the participant’s report) and individual recruitment patterns.

The data were analyzed in four steps. First, weighted estimates (with 95 % confidence intervals [CI]) of the participants’ sociodemographic traits, sexual behaviors, risk perception, and prevention behaviors were computed using RDS Analysis Tool (RDSAT, version 7.1). Second, we exported individualized weights for HIV infection (used as the outcome variable) from RDSAT to SPSS (version 21.0). Third, we conducted (weighted) logistic regression to assess the associations among sociodemographic traits, sexual behaviors, prevention behaviors, risk perceptions, syphilis infection, and HIV status. Univariate odds ratios (OR) and the respective 95 % confidence intervals (CI) were computed and presented in Table 3 if they showed statistical significance (*P* < 0.10). A Chi-square test for linear trend in proportion was used for ordinal variables with more than two categories to investigate trend associations with HIV infection. In the last step, multiple backward stepwise logistic regression models were employed, using those variables with *P* < 0.10 from the univariate analysis as candidates. Statistical significance was defined as *P* < 0.05.

## Results

### Profile of the participants

Of the 424 participants, weighted estimates showed that 38.6 % were 25–34 years old and 27.7 % were 35–44 years old; 57.3 % were unmarried and 36.8 % were married; 16.4 % had attained college education or above; 45.7 % earned an income of less than 3000 Yuan (approximately 470 US$); and 25.5 % had resided in Wenzhou for less than 1 year and 19.6 % for over 10 years; 38.6 % expressed that they were homosexual and 57.0 % that they were bisexual (Table 1). We compared sociodemographic data between this study and the 2013 surveillance study in Wenzhou [19], and found that our participants were less likely to be well educated (39.9 % versus 56.1 % receiving senior high school education and above, *P* < 0.001) and more likely to be older (43.4 % versus 35.6 % over 34 years old, *P* = 0.023).

**Table 1:** Crude and adjusted estimates of socio-demographic characteristics of MSM in Wenzhou (*N* = 424)

	N (Crude %)	Weighted % (95 % CI)*
Age		
≤24	84 (19.8)	20.6 (14.9–28.2)
25–34	156 (36.8)	38.6 (31.8–46.2)
35–44	127 (30.0)	27.7 (20.2–33.8)
≥45	57 (13.4)	13.2 (8.2–18.1)
Marital status		
Unmarried	238 (56.1)	57.3 (50.8–65.1)
Married	154 (36.3)	36.8 (29.2–43.0)
Divorced/widowed	32 (7.5)	5.9 (3.4–9.3)
Education		
Junior high school and below	255 (60.1)	54.8 (46.9–62.2)
Senior high school	114 (26.9)	28.7 (22.8–34.9)
College and above	55 (13.0)	16.4 (11.3–21.9)
Income		
<3000	179 (42.2)	45.7 (38.7–53.2)
3000–3999	118 (30.2)	28.0 (22.0–35.2)
≥4000	127 (27.6)	26.3(20.3–32.0)
Years for residing in Wenzhou		
<1	100 (23.6)	25.5 (19.5–31.6)
1–3	110 (25.9)	25.5(19.5–32.0)
4–10	125 (29.5)	29.4 (22.5–36.7)
>10	89 (21.0)	19.6 (13.6–25.3)
Sex orientation		
Homosexual	175 (41.3)	38.6 (31.8–46.0)
Bisexual	232 (54.7)	57.0 (49.9–64.2)
Other	17 (4.0)	4.4 (1.8–6.8)

*Respondent driven sampling data were adjusted according to the network size and recruitment pattern. CI, confidence interval

Weighted estimates revealed that the most common venue by which participants found sexual partners was websites (42.6 %), parks (31.5 %), the gay bar (13.7 %), the bathhouse (7.3 %), and other venues (4.9 %), respectively (Table 2); 18.2 % of participants had sex for the first time at less than 20 years of age and 32.6 % at 30 years or older.

**Table 2:** Crude and adjusted estimates of sexual behaviors, prevention behaviors, risk perception, and serological results among MSM in Wenzhou (*N* = 424)

	N (Crude %)	Weighted % (95 % CI)*
Places to find sexual partners mostly		
Gay bar	57 (13.4)	13.7 (8.4–18.8)
Bathhouse	33 (7.8)	7.3 (3.2–11.9)
Park	154 (36.3)	31.5 (24.2–39.8)
Internet	162 (38.2)	42.6 (34.8–52.3)
Other	18 (4.2)	4.9 (2.1–7.9)
Age of first homosexual sex		
≤19	85 (20.0)	18.2 (12.3–23.6)
20–29	220 (51.9)	49.3 (42.2–56.5)
≥30	119 (28.1)	32.6 (26.1–40.6)
Anal sex during previous half year		
Number of anal sex partners		
0	38 (9.0)	11.2 (6.7–16.50
1	134 (31.6)	42.0 (35.6–49.8)
≥2	252 (50.4)	46.8 (39.0–52.6)
General condom use during anal sex (*n* = 386)		
Always	174 (45.1)	41.9 (35.1–49.4)
Sometimes	161 (41.7)	43.2 (35.6–49.5)
Ever	51 (13.2)	14.9 (9.6–21.3)
Anal sex with regular partner		
No	198 (46.7)	45.9 (38.9–53.1)
Yes	226 (53.3)	54.1 (46.9–61.1)
Anal sex with casual partner		
No	143 (33.7)	41.6 (35.1–49.5)
Yes	281 (61.3)	58.4 (50.5–64.9)
Anal sex with commercial partner		
No	396 (93.4)	95.5 (93.1–97.9)
Yes	28 (6.6)	4.5 (2.1–6.9)
Anal sex with group		
No	406 (95.8)	97.8 (96.1–99.2)
Yes	18 (4.2)	2.2 (0.8–3.9)
Anal sex after drinking alcohol		
No	382 (90.1)	92.1 (88.3–95.7)
Yes	42 (9.9)	7.9 (4.3–11.7)
Anal sex after using drugs		
No	406 (95.8)	96.5 (94.5–98.7)
Yes	18 (4.2)	3.5 (1.3–5.5)
Oral sex during previous half year		
Number of oral sex partners		
0	76 (17.9)	20.3 (15.2–27.1)
1	131 (30.9)	36.9 (30.3–44.8)
≥2	217(51.2)	42.8 (35.0–48.3)
General condom use during oral sex (*n* = 348)		
Always	69 (19.8)	19.6 (13.9–26.0)
Sometimes	83 (23.9)	22.6 (16.5–32.5)
Never	196 (56.3)	57.8 (47.2–64.7)
Oral sex with regular partner		
No	234 (55.2)	54.5 (47.4–61.9)
Yes	190 (44.8)	45.5 (38.1–52.6)
Oral sex with casual partner		
No	174 (41.0)	46.8 (40.5–54.7)
Yes	250 (59.0)	53.2 (45.3–59.5)
Oral sex with commercial partner		
No	405 (95.5)	97.1 (94.9–99.0)
Yes	19 (4.5)	2.9 (1.0–5.1)
Sex with women during previous half year		
Number of female sex partners		
0	270 (63.7)	58.9 (52.1–65.8)
1	113 (26.7)	31.7 (24.7–38.4)
≥2	41 (9.7)	9.4 (5.8–13.8)
General condom use during sex with women (*n* = 154)		
Always	48 (31.2)	36.9 (22.1–54.4)
Sometimes	34 (22.1)	15.7 (−)
Never	72 (46.8)	47.4 (40.1–71.6)
Sex with regular female partner		
No	309 (72.9)	70.7 (64.0–77.1)
Yes	115 (27.1)	29.3 (22.9–36.0)
Sex with casual female partner		
No	382 (90.1)	89.1 (84.8–93.1)
Yes	42 (9.9)	10.9 (6.9–15.2)
Sex with commercial female partner		
No	396 (93.4)	91.8 (87.1–95.7)
Yes	28 (6.6)	8.2 (4.3–12.9)
Prevention behaviors, risk perception and serological results		
Exposure to HIV prevention during the previous year		
No	183 (43.2)	46.5 (39.8–54.7)
Yes	241 (56.8)	53.5 (45.3–60.2)
Ever HIV testing lifetime		
No	204 (48.1)	51.9 (44.9–60.3)
Yes	220 (51.9)	48.1 (39.7–55.1)
Risk perception for HIV		
Impossible	192 (45.3)	49.2 (41.5–56.6)
Somewhat possible	164 (38.7)	36.8 (29.4–43.6)
Possible	68 (16.0)	14.1 (10.1–19.2)
HIV infection		
Negative	326 (76.9)	77.2 (71.5–83.1)
Positive	98 (23.1)	22.8 (16.9–28.5)
Current syphilis infection		
Negative	372 (87.7)	90.3 (86.4–93.6)
Positive	52 (12.3)	9.7 (6.4–13.6)
HIV and syphilis co-infection		
Negative	401 (94.6)	96.0 (93.5–98.2)
Positive	23 (5.4)	4.0 (1.8–98.2)

*Respondent driven sampling data were adjusted according to the network size and recruitment pattern. CI, confidence interval

### Anal sex during the previous 6 months

Of the 424 participants, weighted estimates indicated that 11.2 % of MSM had not engaged in anal sex with a man, 42.0 % had anal sex with one male partner during the previous 6 months, and 46.8 % had two or more sexual partners (Table 2). With respect to the type of partner, 54.1 % engaged in anal sex with a regular partner, 58.4 % with a casual partner, and 4.5 % with a sex worker. Of the participants, 2.2 % had ever participated in anal sex with a group, 7.9 % had anal sex after drinking, and 3.5 % had anal sex after using drugs. Among MSM who had any type of anal sex during the previous 6 months, 41.9 % always used condoms.

### Oral sex during the previous 6 months

Of the 424 participants, weighted estimates indicated that 20.3 % had never engaged in oral sex with a man during the previous 6 months, 36.9 % had engaged in oral sex with one male partner, and 42.8 % had engaged in oral sex with two or more sexual partners. Regarding the type of partner, 45.5 % engaged in oral sex with a regular partner, 53.2 % with a casual partner, and 2.9 % with a sex worker. Among MSM who had any type of oral sex during the previous 6 months, 19.6 % always used condoms.

### Sex with women during the previous 6 months

Of the 424 participants, weighted estimates indicated that 58.9 % of MSM never engaged in vaginal sex with a woman during the previous 6 months, 31.7 % had sex with one female partner, and 9.4 % had sex with two or more female partners. Regarding the type of partner, 29.3 % engaged in vaginal sex with a regular partner, 10.9 % with a casual partner, and 8.2 % with a female sex worker. Among MSM who had any type of vaginal sex during the previous 6 months, 36.9 % always used condoms.

### Condom use

**Fig. 1:**
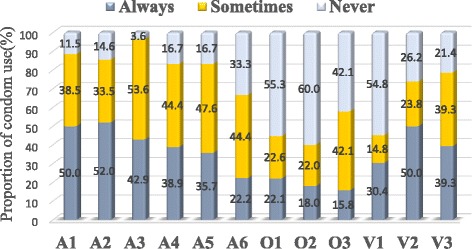
Estimated proportion of condom use during the previous half year. A1 = Anal sex with a regular partner (*N* = 226). A2 = Anal sex with a casual partner (*N* = 281). A3 = Anal sex with a sex worker (*N* = 28). A4 = Anal sex with group partners (*N* = 18). A5 = Anal sex after drinking alcohol (*N* = 42) A6 = Anal sex after using drugs (*N* = 18). O1 = Oral sex with a regular partner (*N* = 190). O2 = Oral sex with a casual partner (*N* = 250). O3 = Oral sex with a sex worker (*N* = 19). V1 = Vaginal sex with a regular female partner (*N* = 115). V2 = Vaginal sex with a casual female partner (*N* = 42). V3 = Vaginal sex with a female sex worker (*N* = 28).

Weighted estimates indicated that the rate of consistent condom use was 50.0 % for MSM who conducted anal sex with a regular male partner, 52.0 % with a casual partner, and 42.9 % with a sex worker (Fig. 1). Of the MSM who conducted anal sex with a group, 38.5 % always used a condom. These rates were 35.7 % and 22.2 % for those who conducted anal sex after alcohol drinking and after drug use, respectively.

Regarding oral sex, the consistent condom use rates were 22.1 %, 18.0 %, and 15.8 % among participants who engaged in oral sex with regular partners, casual partners, and sex workers, respectively. With regard to sex with women, the rates were 30.4 %, 50.0 %, 39.3 % among participants who engaged in vaginal sex with regular partners, casual partners, and sex workers, respectively.

### Prevention behaviors, risk perception and serological results

Over half of the participants (53.5 %) had received HIV-related interventions during the previous year (Table 2), including distribution of condoms, lubricant and education materials, HIV and sexually transmitted infection (STI) counseling, training for prevention of HIV and STIs, and STI checks and treatment. Of the participants, 48.1 % had never been tested for HIV during their lifetime; only 14.1 % thought that they were at risk of HIV.

Among all participants, the weighted prevalence of HIV infection was 22.8 %, that for current syphilis infection was 9.7 %, and that for HIV and syphilis coinfection was 4.0 %.

### Factors associated with HIV infection among MSM

The variables associated with HIV infection in the univariate analysis (*P* < 0.10) are presented in Table 3. *P* values of Chi-square test for linear trend in proportion were also exhibited for ordinal variables with more than two categories. The multivariate analysis revealed that age over 44 versus less than 25 years (AOR = 3.60, 95 % CI = 1.34–9.64), earning an income of 3000–3999 yuan (approximately 470–626 US$) versus less than 3000 yuan per month (AOR = 1.96, 95 % CI = 1.67–3.60), having multiple anal sex partners versus one or no anal sex partners (AOR = 1.93, 95 % CI = 1.15–3.24), awareness versus no awareness of being at risk of HIV (AOR = 3.18, 95 % CI = 1.56–6.48), and current syphilis infection (AOR = 3.01, 95 % CI = 1.44–6.29) were predictors of HIV infection.

**Table 3:** Factors associated with HIV infection among men who have sex with men in Wenzhou

	Univariate (95 % CI)	*P* value	Adjusted OR (95 % CI)	*P* value
Age		0.123*		
≤24	1		1	
25–34	1.61 (0.82–3.19)	0.170	1.59 (0.73–3.47)	0.240
35–44	1.52 (0.74–3.14)	0.258	1.38 (0.62–3.07)	0.428
≥45	2.14 (0.93–4.91)	0.074	3.60 (1.34–9.64)	0.011
Marital status				
Unmarried	1			
Married	1.53 (0.94–2.49)	0.086		
Divorced/widowed	1.40 (0.54–3.65)	0.488		
Education		0.005*		
Junior high school and below	1			
Senior high school	0.80 (0.47–1.37)	0.420		
College and above	0.38 (0.17–0.87)	0.022		
Income per month		0.520*		
<3000	1		1	
3000–3999	1.69 (1.00–2.85)	0.052	1.96 (1.67–3.60)	0.031
≥4000	0.69 (0.37–1.31)	0.259	0.84 (0.41–1.70)	0.621
Years for residing in Wenzhou		0.014*		
<1	1			
1–3	1.06 (0.58–1.95)	0.922		
4–10	0.69 (0.37–1.29)	0.244		
>10	0.41 (0.18–0.89)	0.025		
Number of anal sex partner during previous half year				
0–1	1		1	
≥2	1.96 (1.22–3.14)	0.005	1.93 (1.15–3.24)	0.012
Anal sex with a regular partner				
No	1			
Yes	0.65 (0.41–1.04)	0.073		
Anal sex after using drugs during previous half year				
No	1			
Yes	3.05 (0.99–9.37)	0.051		
Number of oral sex partner during previous half year				
0–1	1			
≥2	1.74 (1.90–2.78)	0.020		
Oral sex with a casual partner during previous half year				
No	1			
Yes	1.62 (1.01–2.61)	0.047		
Risk perception for HIV		0.001*		
Impossible	1		1	
Somewhat possible	1.70 (1.00–2.87)	0.049	1.44 (0.81–2.55)	0.218
Possible	2.86 (1.49–5.47)	0.002	3.18 (1.56–6.48)	0.001
Exposure to HIV prevention during the past year				
No	1			
Yes	0.67 (0.42–1.07)	0.095		
Present Syphilis Infection				
No	1		1	
Yes	2.85 (1.45–5.62)	0.002	3.01 (1.44–6.29)	0.003

*P values of Chi-square test for linear trend in proportion

## Discussion

Our study revealed a striking HIV prevalence of 22.8 % among MSM in Wenzhou, which is much higher than the 7.2 % rate reported in the surveillance data in Wenzhou in 2013 [19]. The RDS method could have resulted in recruiting a more diverse and higher risk group of MSM who are largely hidden from intervention research and service delivery [22]. Use of the RDS method may have helped identify MSM who were inaccessible to surveillance using convenience sampling, and participants were recruited mainly from gay venues and the internet. Furthermore, the incentive offered for participation may have attracted more participants from low socio-economic groups, who are vulnerable to HIV infection [23]. Our study showed that older MSM are more at risk of HIV infection than are young MSM, which is in agreement with a nationwide survey in China [2]. We recruited less-educated and older MSM compared with surveillance data from 2013. Such large differences between this study and local surveillance for HIV prevalence are highly noteworthy. The HIV epidemic reflected in surveillance data is probably greatly underestimated. Thus, it is highly recommended to recruit MSM using powerful methods such as RDS when surveillance or other surveys are conducted. HIV testing must be intensified to detect HIV positive individuals as soon as possible, as the HIV epidemic is already deeply rooted among MSM in this area.

The adjusted HIV infection rates found by other RDS surveys conducted previously are much lower than those reported in this research, such as 6.67 % in Nanjing in 2008 [3], 5.2 % in Guangzhou in 2008 [4], and 8.0 % in Beijing 2009 [5]. Given the rapid increase in HIV prevalence among MSM in China, it is necessary to conduct surveys using RDS and other probability sampling methods to observe the current HIV situation among MSM in various parts of China.

This study provided an overall view of how MSM engaged in different types of anal sex, oral sex with males, and vaginal sex during the previous 6 months, the numbers and types of sexual partners, and how often they used protection during the different forms of sexual intercourse.

During the previous 6 months, 88.8 % of MSM had engaged in anal sex. We found that the rate of consistent condom use was only 42–52 % during sex with regular partners, casual partners, and sex workers. When also considering the high proportions of regular, casual, and multiple sexual partnerships for anal sex, the data indicate a highly risky sexual network that could transmit HIV through anal sex, leading to a reasonable explanation for the high prevalence of HIV infection in this group. Our study reported that 4.5 % of participants engaged in anal sex with sex workers, with a consistent condom use rate of less than 50 %. Although this rate is low, this group should still be given particular attention due to their high-risk behaviors. A study in China showed that male sex workers are more likely to have sex with multiple partners or groups and are more likely to use alcohol or illicit drugs than are other MSM [24].

We reported specifically low rates of consistent condom use among those who engaged in anal sex with a group and after drinking alcohol and using drugs. Although these behaviors are not common, they could lead to risky sexual behaviors, such as having multiple sex partners, casual partners, and partners of unknown or discordant HIV status, more common among MSM [25–27], thus increasing their vulnerability to HIV transmission. Prevention activities, such as condom use and harm reduction, are needed to protect this group.

Engaging in oral sex with males is highly prevalent among MSM; 80 % of the study subjects engaged in oral sex during the previous 6 months—over half with a casual partner and 42 % with more than one partner. Highly prevalence engagement in oral sex was not accompanied by similarly high rates of protection, as consistent condom use when engaging in oral sex in this study was only ~20 %, lower than the rates for anal and vaginal sex. Previous literature has shown that HIV [28–30] and certain STIs, including syphilis [31, 32], can be transmitted via oral sex. Given that condom use with oral sex has not been recommended previously or advised by the Chinese government, public health sectors or MSM communities, it is important to educate MSM regarding the risk of HIV as well as STI transmission during unprotected oral sex and the ability to reduce these risks with condom use.

Many Chinese MSM are currently or eventually will be married due to social and family pressures. Our research indicated that 36.8 % of MSM are married and 41.1 % of MSM had engaged in sex with women during the previous 6 months, consistent with results from a meta-analysis in China [14]. We found that the rate of consistent condom use is ~30 % during sex with a regular female partner, compared with 50.0 % with a casual partner and 39.3 % with a sex worker. This is consistent with other Chinese studies showing that MSM who engaged in bisexual behavior had a higher rate of unprotected sex with stable female partners and were less likely to take part in preventive behaviors [15, 33]. This could be due to the desire to have children among MSM and their stable female partners (perhaps due to the pressure of traditional Chinese culture), concern that condom use would cause distrust in their female partner, or lack of awareness of their own risk of HIV infection or the risk of transmitting HIV to their female partner. On the other hand, female partners may not be aware of their male partner’s sexual practices and their own consequent risk of contracting HIV. Other Chinese studies have reported that low condom use with female partners was common in HIV-positive MSM, showing that HIV-positive MSM did not take action to protect their female partners [16, 33]. The combination of engaging in sex with regular female partners, low rates of consistent condom use, and high HIV infection rates places the girlfriends and wives of MSM in Wenzhou at high risk of HIV infection. Approximately 10 % of our participants engaged in sex with a casual female partner and 10 % with a sex worker during the past 6 months, and 40 ~ 50 % of these encounters did not involve consistent protection. Furthermore, 10 % of MSM also have sex with multiple female partners, resulting in serious concern for HIV transmission by these MSM and further increasing the prevalence of HIV among the heterosexual population. One Chinese study already indicated that the substantially increasing HIV prevalence among MSM led to an increase in HIV incidence among female partners of bisexual MSM [34].

We reported that approximately half of MSM had never been tested for HIV during their lifetime. That rate is higher than those in Beijing and Chongqing where one-third of MSM have never been tested for HIV [35, 36]. Further, we reported that 47 % of the participants were not exposed to any HIV intervention measures. HIV testing and related interventions have been promoted among MSM by local CDCs and community-based MSM organizations in Wenzhou over the past decade, but these results indicated deficiencies in the provision and utilization of these services. HIV testing and related intervention in Wenzhou may mainly reach only those who are active in MSM communities, and those who are deeply hidden were rarely reached. The various barriers for MSM to seek these services should also be taken into consideration fully, while promoting HIV testing and delivering prevention services. The following measures may be taken to broaden HIV testing and behavioral intervention among MSM in Wenzhou. First, RDS could be employed as a method to identify those MSM overlooked, though it is mainly used as sampling method. Second, the social networks of MSM could be used for education and intervention [37], especially internet-based social networks, such as the Blued software that is widely used by Chinese MSM. Our results indicated that MSM in Wenzhou use the internet as their primary source of finding sexual partners, which is consistent with other surveys in China [2, 17].

Although HIV has always been highly prevalent among MSM, our study participants were generally under the impression that they had low or no chance of contracting HIV [38]. Our study reported that only 14.1 % of MSM perceived that they were at risk of HIV. This result clearly did not match the highly risky sexual behaviors they engaged in and is not proportional to the actual level of HIV infection in this population. A qualitative study indicated that despite most MSM being aware of the high prevalence of HIV infection among their population, they still thought the chance of HIV infection was rare [39]. Our finding emphasizes the need to enhance risk awareness among MSM in Wenzhou through targeted interventions. HIV risk perception was related to HIV infection in the multivariate analysis. This association may imply that those MSM who perceived themselves to be at risk of HIV infection believed that they engaged in greater levels of risky behavior more likely to result in HIV infection than those of other MSM. A high perceived HIV risk was associated with many of the established risk factors for transmission of HIV and was indeed positively associated with HIV infection [38, 40]. MSM who are perceived to be at high risk should be targeted for HIV prevention efforts to modify their risky sexual practices.

We estimated the prevalence of syphilis to be 9.7 %. Syphilis infection has shown a relationship to HIV infections among MSM [41, 42], which was confirmed in this study. The incidence of syphilis infection in HIV-positive MSM is high [43, 44]. Syphilis co-infection among HIV-positive subjects was associated with significant increases in viral load and significant decreases in CD4 cell counts [45, 46]. Our findings demonstrate that prompt action for prevention, screening and treatment of syphilis should be a priority among both HIV-positive and -negative MSM. Meanwhile, because of the high prevalence of HIV among MSM in this area, and a large number of HIV-positive MSM aware of their HIV status continue to engage in unprotected anal, oral and vaginal sex [16, 43, 44], effective intervention strategies such as anti-retroviral treatment of all HIV-positive people should be initiated immediately to reduce the viral load and prevent HIV transmission in the community [47, 48],

Our study has several limitations. We did not collect information on the roles of anal and oral sex (insertive vs. receptive), which could have different effects on HIV and STI transmission. As this was a cross-sectional study, we could not establish the causality of different sexual behaviors and other variables on HIV infection; therefore, a longitudinal study is needed to further explore variables associated with HIV infection among MSM. Questions regarding sexual behavior may be sensitive for some participants, such that they may give socially desirable responses resulting in information bias, although interviewers underwent intensive training to minimize this effect. Finally, the incentive reward offered by the RDS method may attract more low-income people to participate in the study, potentially limiting the representativeness of the sample, although RDS is effective in accessing hard-to-reach populations [23]. Despite these limitations, this is the first study to investigate the prevalence of various sexual behaviors among MSM in China using RDS and fills an important gap in the literature in Zhejiang province. Findings from this study will be of value in influencing future studies and in developing targeted risk reduction interventions.

## Conclusion

We found that risky sexual behaviors for HIV and STI transmission were extremely common among MSM in Wenzhou, including having multiple sexual partners, engaging in commercial and casual sex, and lack of condom use during anal sex, oral sex with men, and sex with women. Together these behaviors have established multiple sexual networks that have already fueled and could continue to fuel HIV and STI epidemics. Given the striking prevalences of HIV and syphilis and the interaction between the two, these sexual networks are extremely risky. HIV transmission will probably continue to increase, resulting in greater and greater transmission among MSM and their male and female partners if these networks persist. These findings demonstrate a profound urgency for effective interventions, such as HIV risk education, condom promotion, frequent HIV testing, timely diagnosis and treatment of STIs, early initiation of antiretroviral therapy at the population level, and broadening the coverage of these services to slow the rapid spread of HIV in Wenzhou.

## Additional file

Additional file 1:
**Questionnaire survey for Health of comrades (MSM).** (DOCX 49 kb)

## Abbreviation

HIV: Human immunodeficiency virus
MSM: Men who have sex with men
RDS: Respondent-driven sampling
CDC: Center for Disease Control and Prevention
ELISA: Enzyme-linked immunosorbent assay
STI: Sexually transmitted infection
