# Supplementary material for: Risky sexual networks and concentrated HIV epidemics among men who have sex with men in Wenzhou, China: a respondent-driven sampling study
Source: BMC Public Health. 2015 Dec 16;15:1246. doi: 10.1186/s12889-015-2591-7 (PMC4682249; doi:10.1186/s12889-015-2591-7)
Supplement: Additional file 1: — Questionnaire survey for Health of comrades (MSM). (DOCX 49 kb) [file 12889_2015_2591_MOESM1_ESM.docx]

ID code：

Coupon code：

**Questionnaire survey for Health of comrades (MSM)**

Name or alia of participant:

Investigated city：

Investigation agency：

November,2013

**Participant inclusion and exclusion**

| **S1** | Birthday： month year | | |
| --- | --- | --- | --- |
|  | **S11** | If age≥14 years old？ **①**Yes **②**No **(not included in this survey)** |  |
| **S2** | Recent residing address： District City，residing time year(s)**（less than 1 year should be converted into decimal）** | | |
|  | **S21** | If you reside in recent place ≥3 months？ **①**Yes **②**No **(not included in this survey)** |  |
| **S3** | If you have oral or anal sex with male sexual partner(s) in the past year **①**Yes **②**No **(not included in this survey)** | |  |

**A. Basic information**

| **A1** | Marital status：①Cohabitation(with female) ②Cohabitation(with male) ③Single ④Married with spouse  ⑤Divorced or windowed |  |
| --- | --- | --- |
| **A2** | Educational level:   1. Primary school or below **②**Junior high school **③Senior h**igh school **④**College or above |  |
| **A3** | Occupation：   1. Employee（Restaurant/hotel/beauty salon/property logistics and other service industries） **②**Company employees **③**Businessman **④**Government agency/institution staff **⑤**Worker（Construction/manufacturing, etc） **⑥**Student **⑦**Farmer **⑧**Retired **⑨**Unemployed **⑩**Others（note） |  |
| **A4** | Monthly income(RMB:yuan)？   1. No income **②**<1000 **③**1000-1999 **④**2000-2999 **⑤**3000-3999 **⑥**≥4000 |  |
| **A5** | Medical insurance（Single or multiple choices）？  ①[New](javascript:void(0);) [Rural](javascript:void(0);) [Co-operative](javascript:void(0);) [Medical](javascript:void(0);) [System](javascript:void(0);) ②The medical insurance for urban employees ③The [medical](javascript:void(0);) [insurance](javascript:void(0);) [for](javascript:void(0);) [urban](javascript:void(0);) [residents](javascript:void(0);) ④The commercial insurance ⑤No |  |
| **A6** | Self-reported sex orientation？   1. Homosexual ②Heterosexual ③Bisexual ④Unsure |  |
| **A7** | Venues to seek for sexual partners:   1. Gay Bars ②Gay Bathhouse ③Park for MSM ④Internet **⑤**Others（note） |  |
| **A8** | Locally, how many MSM do you know about in your circle of friends_____?**[Knowing about** **means you will be able to know the other face, know the name or alias, nicknames, and contact information, and can be in touch with the other in a month. Give some time to the participants for thinking carefully]** | |
| **A9** | The relationship with the male who recommended you to participate in this program:   1. The past sexual partners**（before 6 months）** **②**The sexual partners in the past 6 months **③**Good friends   **④**Common friends **⑤**Acquaintances **⑥**Strangers |  |
| **A10** | Self-perceived status of physical health about yourself？ **①**Very well **②**Well **③**Normal  **④**Not well **⑤**Very bad |  |

**B. Sexual behavioral characteristics and sexual partner’ network**

| **B1** | Age of sexual debut with a man: years old | | | | | | | |
| --- | --- | --- | --- | --- | --- | --- | --- | --- |
| **B2** | Number of anal sex partners with men in the past **6** months________**（If there is no, jump to B4）** | | | | | | | |
| **B3** | Frequency of condom use engaging in **anal sex** with **male** sexual partners in the past **6** months？ | | | | | | | |
|  | **B31** | Anal intercourse with regular male sexual partners：**①**Never **②**Sometimes **③**Always **④**There was no such sexual activity | | | | | |  |
|  | **B32** | Anal intercourse with casual male sexual partners：**（Neither commercial or regular）**：**①**Never **②**Sometimes **③**Always **④**There was no such sexual activity | | | | | |  |
|  | **B33** | Anal intercourse with male sex workers：**①**Never **②**Sometimes **③**Always **④**There was no such sexual activity | | | | | |  |
|  | **B34** | Group anal intercourse： **①**Never **②**Sometimes **③**Always **④**There was no such sexual activity | | | | | |  |
|  | **B35** | Anal intercourse after drinking alcohol: **①**Never **②**Sometimes **③**Always **④**There was no such sexual activity | | | | | |  |
|  | **B36** | Anal intercourse after using drugs： **①**Never **②**Sometimes **③**Always **④**There was no such sexual activity | | | | | |  |
| **B4** | Number of oral sex partners with men in the past **6** months**（If there is no, jump to B6）** | | | | | | | |
| **B5** | Frequency of condom use engaging in oral sex with men in the past **6** months？ | | | | | | | |
|  | **B51** | Oral sex with regular male sex partners： **①**Never **②**Sometimes **③**Always **④**There was no such sexual activity | | | | | |  |
|  | **B52** | Oral sex with casual male sex partners：**（Neither commercial or regular）**：**①**Never **②**Sometimes **③**Always **④**There was no such sexual activity | | | | | |  |
|  | **B53** | Oral sex with male sex workers：**①**Never **②**Sometimes **③**Always **④**There was no such sexual activity | | | | | |  |
| **B6** | Please recall the male sexual partner in the recent 1 year one by one , you don't need to tell their names, but in order to help you recall this information, you can give them the number or use code (**Please list them according to frequency of sex from more to less in sequence. List all sex partner if you have less than 5 partner; list just 5 partner if you have more than 5 partner**) | | | | | | | |
|  | **Sexual partner’ characteristics** | | **No.1** | **No.2** | **No.3** | **No.4** | **No.5** | |
|  | **B61** | **Relationship between you and male sexual partners：**  **①** Regular male sexual partners **②**Casual male sexual partners**（Neither commercial nor regular）③** Commercial male sexual behavior  **④**Other_ |  |  |  |  |  | |
|  | **B62** | **Age of the sexual partner：①**<20 **②**20～ **③**30～ **④**40～ **⑤**≥50 |  |  |  |  |  | |
|  | **B63** | **Frequency of sexual behavior：**  **①**≥twice every week **②**4～7times every month **③**Once to 3 times every month **④**<once every month |  |  |  |  |  | |
|  | **B64** | **HIV-positive status of sexual partners：①**Positive **②**Negative **③**Unclear |  |  |  |  |  | |
| **B7** | Age of vaginal sexual debut with a woman: **①** years old **②** There was no sex with women**（Jump to C1）** | | | | | | |  |
| **B8** | Number of vaginal **female** sexual partners in the past **6** months________**（If there is no, jump to C1）** | | | | | | | |
| **B9** | Frequency of condom use engaging in **vaginal sex** with **female** sexual partners in the past **6** months？ | | | | | | | |
|  | **B91** | **Regular** female sex partners: **①**Never **②**Sometimes **③**Always **④**There was no such sexual activity | | | | | |  |
|  | **B92** | Casual female sex partners （**Neither commercial nor regular**）：**①**Never **②**Sometimes **③**Always **④**There was no such sexual activity | | | | | |  |
|  | **B93** | Female sex workers： **①**Never **②**Sometimes **③**Always **④**There was no such sexual activity | | | | | |  |

**C. Measurement of mood（****CES-D）**

| The following is your possible feelings or acts, point out all kinds of feelings and behavior happened to you in  the last week according to your actual situation, please fill in the corresponding number：   1. Almost never(less than 1 day) ②Sometimes (1-2 days) ③Often (3-4 days) ④Most time (5-7 days) | | |
| --- | --- | --- |
| **C1** | The things which don’t bother me at ordinary times are always bothering me. |  |
| **C2** | I don't want to eat and I have got a bad appetite. |  |
| **C3** | I think that I can't get rid of depression mood even with the help of family and friends. |  |
| **C4** | I think I'm just as well as others. |  |
| **C5** | I can't concentrate on my attention. |  |
| **C6** | I feel suppressed. |  |
| **C7** | I don't think I do everything easily. |  |
| **C8** | I feel hopeful about the future. |  |
| **C9** | I think my life was a failure. |  |
| **C10** | I feel scared. |  |
| **C11** | My sleep is not [smooth](javascript:void(0);) or [steady](javascript:void(0);). |  |
| **C12** | I feel happy. |  |
| **C13** | I speak less than usually. |  |
| **C14** | I feel lonely. |  |
| **C15** | People are not friendly enough. |  |
| **C16** | I enjoy my life. |  |
| **C17** | I cried once. |  |
| **C18** | I feel bad. |  |
| **C19** | I don't think people like me. |  |
| **C20** | I can't get into the state. |  |

**D. Behavior and social cultural characteristics**

| **D1** | Do you smoke? **[Smoking means smoking one or more cigarettes every day for more than a year or smoking more than 300 cigarettes in the short term (3 months or less**)**]**   1. Current smoker **②**Former smoker**(Having never smoked in the past 6 months)**   **③**Never smoke **(Jump to D2)** | |  |
| --- | --- | --- | --- |
|  | **D11** | When did you start to smoke？Age_____ (or _____year).  Accumulated ____years of smoking excluding time not to smoke up to now. | |
|  | **D12** | **Number of cigarettes you smoke on average every day. (Generally a pack of cigarettes is 20 cigarettes.)________** | |
| **D2** | Do you drink**[Drinking refers to drinking alcohol intake of 100 grams per week]**   1. Drinking now **②**Drinking before**(No drinking in the past six months)** **③**Never drinking**(Jump to D5)** | |  |
|  | **D21** | From ____aged years old (or ___years), you started to smoke.  Accumulated ____years of drinking excluding time not to drink up to now. | |
|  | **D22** | ______Times of drinking a week on average, drinking intake volume every time generally：   1. Beer___ bottles(___ml) **②**Red Wine bottles（ ml） **③**Liquor liang（ degree wine）   **④**Yellow wine liang（ degree wine） **⑤**Others， wine**(What kind)** ml（ degree wine） | |
| **D3** | If you are drunken in the past year? **①**Often **②**Sometimes **③**Never**(Jump to D5)** | |  |
| **D4** | If you want or need to reduce your drink in the past 1 year? **①**Yes **②**No | |  |
| **D5** | If you have used drugs more than you need in the past 1 year(**including Psychotropic drugs, such as sedative hypnotics, caffeine, wheat carlin, LSD, and narcotic drugs, such as opiates, cocaine, cannabinoids, etc**)?   1. Often **②**Sometimes **③**Never**(Jump to D7)** | |  |
| **D6** | If you want or need to reduce your drug use? **①**Yes **②**No | |  |
| **D7** | Have you ever suicide tendency？ **①**Never **②**Sometimes **③**Often | |  |
| **D8** | If you suffered from a male sexual partner violence?（**sexual violence includes physical, sexual or mental violence**）   1. Never **②**Sometimes **③**Often | |  |
| **D9** | If you suffered from sexual abuse in your childhood? **①**Yes **②**No | |  |
| **D10** | What impact do you think the current social perception about MSM will have on your sexual behavior?   1. Increasing protected sex with a male**②**Increasing protected sex with a female **③**Increasing unprotected sex with a male **④**Increasing unprotected sex with a female **⑤**No impact | |  |
| **D11** | Self-perceived possibility of HIV infection: **①**Probable **②**Possible **③**Unlikely **④**Impossible | |  |
| **D12** | Estimate of HIV prevalence among MSM now: **①**≤5% **②**6-10% **③**11-15% **④**16-20% **⑤**≥21% | |  |
| **D13** | **Frequency of exposure to HIV prevention in the past year（**including distribution of condoms, lubricant and education materials, HIV and sexually transmitted infection counseling, training for prevention of HIV and STIs, and STI checks and treatment）   1. Never **②**1~2 times a year **③**2~5 times half year **④**1~3 times a month **⑤**1~4times a month | |  |

**E. The history and intention of HIV testing**

| **E1** | Have you ever undertaken an HIV testing?**①**Yes **②**No**（Jump to E6）** | |  |
| --- | --- | --- | --- |
| **E2** | Times of HIV testing in the past year:__________ | | |
| **E3** | Date of the recent HIV testing: mm/yyyy | | |
| **E4** | The type of the recent HIV testing:   1. VCT (Voluntary Counseling And Testing) **②**Testing service provided by CDC **③**Blood/Blood products transfusion **④**Blood donation **⑤**Physical examination for employment   **⑥**Premarital physical examination **⑦**Hospitalized examination **⑧**Physical examination for enrollment **⑨**Other（Please specify） | |  |
| **E5** | Do you have the habit of regular HIV testing？ **①**Yes **②**No**（Jump to E6）** | |  |
|  | **E51** | The reason of regular HIV testing**（Single or multiple choices）**:   1. Health Surveillance  **②**Frequent high-risk sexual behaviors **③** Distrusting the regular sexual partner **④**The regular sexual partner is positive **⑤** To start a new partnership with the other **⑥**Other（Please specify） |  |
|  | **E52** | How long do you uptake an HIV test?（**Jump to F1 after finishing the question）**   1. 3 months **②**Half year **③**One year **④**Other（Please specify） |  |
| **E6** | Are you willing to accept regular testing for HIV? **①**Yes**（Jump to F1）** **②**No | |  |
|  | **E61** | If no, the reason is______（**Single or multiple choices）**   1. Don't know where to test ②It is difficult to take a test on a regular time due to work or the living habits ③Worry about the positive test results   **④**It is not convenient to go to the test place **⑤**Fear of the discrimination after personal identity exposure **⑥**There is no high-risk behavior  ⑦I am very safe because I only have regular sexual partners **⑧**Other（Please specify） |  |

**F. Cognition antiviral treatment and intention of early treatment**

| **F1** | Did you know about antiviral treatment for HIV/AIDS? **①**Very well **②**Generally **③**A little **④**Almost no |  |
| --- | --- | --- |
| **F2** | Regarding antiviral treatment, you may be more attention to what aspects (**multiple choices if possible**)?   1. Medicine effect ②Side effect ③[Medication](javascript:void(0);) [compliance](javascript:void(0);)   ④ Privacy protection **⑤**Convenience of taking medicine **⑥**drugs resistance  **⑦**Financial burden **⑧**Other（Please specify） |  |
| **F3** | Do you think if it is necessary for HIV-positive people to accept early antiviral treatment? **①**Yes **②**No |  |
| **F4** | Whose suggestions do you think can mostly promote HIV-positive people to accept the early antiviral treatment?   1. Doctor ② CDC staff **③**MSM volunteer **④**Patient **⑤**Other（Please specify） |  |

**This questionnaire survey now is over! Thank you for your participation! Welcome to put forward your precious comments for this survey!**

**­**
